# Investigation of the indoor ^222^Rn and ^220^Rn levels in the residential environment and estimation of the annual effective radiation dose for ordinary residents

**DOI:** 10.1371/journal.pone.0253463

**Published:** 2021-06-24

**Authors:** Xiaohong Li, Hongyang Ke, Chuan Ouyang, Xiaoli Yu, Yumei Liu, Fei Wang, Wanwei Li

**Affiliations:** 1 School of Public Health, Weifang Medical University, Weifang, Shandong Province, China; 2 Public Health Testing Center, School of Public Health and Management, Weifang Medical University, Weifang, Shandong Province, China; 3 Weifang Key Laboratory of Health Inspection and Quarantine, Weifang, Shandong Province, China; 4 Experimental Demonstration Teaching Center of Public Health, School of Public Health, Weifang Medical University, Weifang, Shandong Province, China; Institute for Advanced Sustainability Studies, GERMANY

## Abstract

To evaluate the health risk of radon and its progeny, a large amount of accurate monitoring data is needed according to the theory and practice of health risk assessment. However, the indoor radon levels in different regions in China and worldwide reveal temporal and spatial variations. In addition, the residents living in different areas follow distinct living modes. Therefore, it is recommended and accepted by many researchers to detect the radon level in local areas and subsequently conduct health risk assessments based on local detection data. In this study, 21 bedrooms of households in Weifang city were selected, and the indoor ^222^Rn and ^220^Rn levels were detected with RAD7 radon detector in winter, while the annual effective radiation dose was calculated for ordinary residents in Weifang city. Our investigation showed that the 24- and 12-hour average levels of ^222^Rn were 35.7±15.2 Bq/m^3^ and 36.2±15.8 Bq/m^3^, respectively. The 24- and 12-hour average levels of ^220^Rn were 30.4±12.3 Bq/m^3^ and 22.4±11.6 Bq/m^3^, respectively. There were significant differences in the average levels of ^222^Rn and ^220^Rn between floors. The estimated annual effective radiation dose received by ordinary residents in Weifang city was 1.7193 mSv, of which 0.9479 mSv originated from ^222^Rn and its progeny and 0.7714 mSv originated from ^220^Rn and its progeny, accounting for 55.1% and 44.9%, respectively, of the total dose. Our findings suggest that ^220^Rn should not be ignored by local residents in Weifang city, and more attention should be paid to ^220^Rn in future research.

## 1. Introduction

Radon is a colorless, odorless natural radioactive gas. Radon and its daughters occur in all living and working environments. Under normal circumstances, the natural radiation dose received by humans is approximately 2.4 mSv per year, and 50% of the total dose originates from exposure to radon and its daughters. The No. 50 report released by the International Commission on Radiological Protection (ICRP) estimates that 10% of lung cancer cases can be attributed to exposure to radon and its progeny, which is one of the 19 carcinogens identified by the World Health Organization (WHO). Radon has been classified as a class I carcinogen by the International Agency for Research on Cancer (IARC).

There were large-scale radon surveys conducted by the former Ministry of health in 1980-1990S and 2001-2004S. To better understand the baseline level of soil radon in China, the former Ministry of Construction conducted the first nationwide soil radon survey from 2003 to 2005. This survey revealed that the soil average radon concentration in China was 7300 Bq/m^3^, and the first soil radon distribution map of China was provided. This survey marked the first step in the study of radon in China. In regard to indoor radon, research started with a nationwide investigation referred to as China Indoor Radon Research from 2007 to 2010, which was jointly organized by the Henan Province Academy of Building Sciences and the Building Indoor Radon Professional Committee of the Chinese Radiation Protection Society. This investigation comprised the main task of the research content on the key technologies of indoor radiation pollution control and improvement in buildings, as part of the science and technology support plan of the Eleventh Five-Year Plan of China. This investigation indicated that the average indoor radon level in China was approximately 36.1 Bq/m^3^. Through these two large-scale studies, our understanding of indoor radon in China has been greatly improved.

Radon is a natural radioactive gas with three natural radioisotopes, ^222^Rn, ^220^Rn and ^219^Rn, and their half-lives are 3.825 days, 55.6 seconds and 4.0 seconds, respectively. Because of the short half-lives of ^220^Rn and ^219^Rn, they have often been ignored in previous studies, so radon often refers to ^222^Rn. However, with the advent of in-depth radon research, in the early 1990s, a survey conducted in Japan found that the ^220^Rn concentration in houses with a traditional soil structure exceeded that of ^222^Rn [1]. An investigation performed in China also showed that the ^220^Rn level in houses with a traditional soil structure in Pingliang, Gansu Province, was 5–10 times that of ^222^Rn [2]. These findings challenge the long-standing misconception that ^220^Rn in residential environments can be ignored. Although the half-life of ^220^Rn is very short, it may release 6.29 MeV of alpha particles upon decay and produce the key decay daughters of ^212^Pb and ^212^Bi, of which their half-life is much longer than that of ^222^Rn progeny. Subsequently, the radiation dose attributed to ^220^Rn could be highly notable. A United Nations Scientific Committee on the Effects of Atomic Radiation (UNSCEAR) report released in 2000 estimates that ^220^Rn and its progeny account for 20.5% of the radon dose and points out that the dose conversion factor of ^220^Rn progeny may be twice the value recommended by the UNSCEAR or higher, and the contribution of ^220^Rn to the radon dose may be equivalent to or even exceed that of ^222^Rn [3]. Additionally, the ^232^Th content in Chinese soil is higher than the worldwide average level, and the ^222^Rn and ^220^Rn concentrations in residential environments may reach relatively high levels [4].

Approximately 80% of people’s lives is spent indoors, and the residential environment accounts for a large part of the indoor environment. Accordingly, there exists a close relationship between human health and living environment. It has been estimated that 6600–24000 lung cancer deaths per year may be related to indoor radon in the United States in the late 1980s [5]. Indoor radon accounts for nearly half of all radiation exposure among the general population [6]. Research results from a combined analysis of 7 North American case-control studies have provided direct evidence of an association between residential radon and lung cancer risk [7]. Under the same exposure conditions, the health risk originating from long-term low-dose exposure is higher than that originating from short-term high-dose exposure [8]. A comprehensive study integrating two large-scale studies in China has indicated that long-term radon exposure at levels encountered in many homes increases the lung cancer risk [9]. Experimental studies have also shown direct damage of cellular DNA after the traversal of cultured mammalian cells by single alpha particles and have provided direct evidence of the potential for radon carcinogenicity at low exposure levels [10, 11]. Hence, indoor low-level radon exposure is common and has become a major public health concern.

Any health risk assessment relies on the monitoring of the exposure level. In terms of the lung cancer risk caused by indoor radon exposure, monitoring data are also needed. It must be noted that regional differences should be considered when determining radon exposure levels so that health risk assessments can be carried out more accurately. Weifang is the third largest city in Shandong Province, with a population of over 7 million people. With rapid economic development, people’s residential environment has been greatly improved, and high-rise buildings have become the main architectural form in residential communities. However, there is a lack of basic data on indoor radon in high-rise buildings in Weifang, in addition to a lack of health risk assessment data of radon for ordinary residents. How high is the radon concentration in the residential environment in Weifang? What is the annual effective radiation dose received by ordinary residents in Weifang? These questions should be addressed at present. Against the background of the notable improvement in residential conditions, a nationwide indoor radon survey is urgently needed in China.

In this study, three typical residential communities in Weifang city were selected as research sites, and 21 bedrooms were selected as sampling points. Indoor ^222^Rn and ^220^Rn levels were detected with RAD7 radon detector, and the annual effective radiation dose for ordinary residents in Weifang city was calculated. This study could provide a primary reference for the health risk assessment of radon exposure for ordinary local residents.

## 2. Method

### 2.1 Description of the 21 selected bedrooms in the three residential communities

Three residential communities built 3–5 years ago in Weifang city were randomly selected. The buildings were all constructed of steel, concrete and cement. They were usually built with a frame structure, which is also the common construction mode of residential buildings in China. Twenty-one households in three residential communities were selected as sampling points. Among them, 11, 3 and 7 households occurred on the first, sixth and twelfth floors, respectively. Each selected household contained three members, including parents and one child, which is also the common family structure in China. The members of the 21 households followed similar living habits, namely, they went to work or school at approximately seven or half past seven in the morning and returned home at approximately seven or half past seven in the afternoon. No one was home during work and school hours. Hence, family members only spent 12 hours in their respective households. The floors of the 21 selected households contained ceramic tiles, and the heating mode was floor heating, while the homeowners were asked not to close their bedroom door at night when they went to bed to ensure air flow uniformity in the whole household during the detection period. At night, the windows of the residence remained closed to facilitate heat preservation because the detection period occurred during the local winter season. All the rooms of the 21 households were in normal use during the monitoring period.

### 2.2 Monitoring method

One bedroom in each household was selected as a sampling point. The indoor ^222^Rn and ^220^Rn concentrations in the 21 bedrooms were detected with RAD7 radon detector (Durridge Co., USA). The RAD7 radon detector is a highly versatile instrument that constitutes the basis of a comprehensive radon measurement system. The RAD7 radon detector was placed near the center of each selected bedroom, approximately 3–4 feet above the floor, thereby avoiding walls, vents, windows, and direct sunlight. During detection, the internal relativity humidity was controlled to lower than 5% by using a desiccant. The desiccant was replaced every 7 to 14 days, depending on the indoor relative humidity. Before instrument start-up, a test purge program was performed outdoors for 10–20 minutes to purify the sample chamber and remove any background radon. The detection cycle was set to 1 hour, and the detection recycling interval was set to 24 hours. The auto-mode was selected. A 24-hour test was conducted every 1–2 days from October 28, 2019, to December 28, 2019.

### 2.3 Estimation of the annual effective radiation dose

The annual effective radiation dose of indoor ^222^Rn and ^220^Rn exposure was calculated with the equation below retrieved from the UNSCEAR [5].

$$D_{R}(mSv/y)=(0.17+9\times F_{R})\times C_{R}\times 8760\times H\times 10^{‐6}$$ (1)

$$D_{T}(mSv/y)=(0.11+40\times F_{T})\times C_{T}\times 8760\times H\times 10^{‐6}$$ (2)

where D_R_ is the annual effective radiation dose of ^222^Rn exposure and D_T_ is the annual effective radiation dose of ^220^Rn exposure. The values of 0.17 and 9 are dose conversion factors for the concentrations of ^222^Rn and its progeny, respectively, while the values of 0.11 and 40 are dose conversion factors for the concentrations of ^220^Rn and its progeny, respectively, in nSv [12]. F_R_ and F_T_ are equilibrium factors for ^222^Rn and ^220^Rn and their progeny, respectively, and worldwide, typical F_R_ and F_T_ values are 0.4 and 0.1, respectively. C_R_ and C_T_ are the ^222^Rn and ^220^Rn concentrations, respectively, in Bq/m^3^, and 8760 is the number of hours in a year. H is the occupancy factor, and the values of H are 0.50 (12 h/24 h), 0.33 (8 h/24 h) and 0.17 (4 h/24 h) in indoor residential environments, indoor working environments and outdoor environments, respectively. A multiplication factor of 10^−6^ is adopted to convert nSv into mSv.

### 2.4 Data processing and statistical analysis

The 24- and 12-hour average ^222^Rn and ^220^Rn concentrations determined through continuous monitoring were expressed as the arithmetic mean and standard deviation (M±SD). The Kruskal-Wallis H test was applied to compare the three groups, and the Mann-Whitney U test was applied to compare any two groups. These statistical methods were performed in SPSS 21.0. A *p* value less than 0.05 (*p*<0.05) was considered statistically significant.

## 3. Results

### 3.1 Raw indoor ^222^Rn concentrations

The 24- and 12-hour ^222^Rn average concentrations continuously measured in the 21 households are presented in Table 1. During the detection period, the maximum ^222^Rn level in the principal bedrooms reached approximately 85.0 Bq/m^3^. The minimum ^222^Rn value was approximately 3 Bq/m^3^. The Mann-Whitney U test showed that there was no significant difference between the 24- and 12-hour average ^222^Rn concentrations (U = 62201, *p* = 0.645).

**Table 1:** Average ^222^Rn level in the 21 bedrooms (Bq/m^3^).

Number of rooms	Monitoring days	Location	Range	24-h average level	12-h average level
1	30	12^th^ floor	9.2–39.5	20.4±6.9	20.0±8.5
2	25	12^th^ floor	12.3–43.0	28.0±13.2	21.7±10.1
3	26	12^th^ floor	10.5–43.2	24.8±11.1	20.7±9.7
4	25	12^th^ floor	14.0–42.4	27.8±14.7	21.0±12.6
5	26	12^th^ floor	3.0–41.7	25.5±11.1	19.9±9.2
6	26	12^th^ floor	9.0–41.2	26.5±10.0	30.8±9.0
7	24	12^th^ floor	10.6–55.9	29.2±12.4	29.5±11.3
8	30	6^th^ floor	25.1–63.6	37.8±11.2	42.5±10.1
9	25	6^th^ floor	20.0–53.1	30.8±13.2	27.9±14.0
10	26	6^th^ floor	28.2–67.0	34.0±12.4	40.1±10.2
11	28	1^st^ floor	40.9–72.3	49.7±13.0	52.3±11.8
12	31	1^st^ floor	21.4–66.5	49.3±14.8	54.0±9.0
13	29	1^st^ floor	28.2–63.6	43.1±13.2	41.5±13.8
14	28	1^st^ floor	20.0–62.1	41.5±10.3	41.3±10.3
15	26	1^st^ floor	21.6–56.4	37.2±15.7	41.6±14.4
16	24	1^st^ floor	16.0–70.0	35.3±11.0	39.0±7.7
17	27	1^st^ floor	15.2–66.0	45.3±16.2	45.8±16.9
18	28	1^st^ floor	23.1–58.4	37.5±16.3	34.2±14.1
19	26	1^st^ floor	22.5–70.0	40.2±13.4	44.3±13.2
20	28	1^st^ floor	27.2–62.8	40.1±15.8	45.7±16.2
21	30	1^st^ floor	26.1–85.0	45.1±13.6	46.3±15.4
Total average level			3.0–85.0	35.7±15.2	36.2±15.8

### 3.2 Raw indoor ^220^Rn concentrations

The inert-gas isotope ^220^Rn is a gaseous member of the natural decay chain starting from ^232^Th. It is transferred to the atmosphere via diffusion from the uppermost few centimeters of soil. The 24- and 12-hour average ^220^Rn concentrations continuously measured in the 21 households are listed in Table 2. During the detection period, the maximum ^220^Rn value in the bedrooms was approximately 221.0 Bq/m^3^. The minimum ^220^Rn value reached approximately 4 Bq/m^3^. The Mann-Whitney U test indicated that there was a significant difference between the 24- and 12-hour average ^220^Rn concentrations (U = 56050.5, *p* = 0.008).

**Table 2:** Average ^220^Rn level in the 21 bedrooms (Bq/m^3^).

Number of rooms	Monitoring days	Location	Range	24-h average level	12-h average level
1	30	12^th^ floor	5.0–49.2	21.5±10.4	21.5±10.2
2	25	12^th^ floor	10.1–44.5	22.4±9.0	26.0±8.7
3	26	12^th^ floor	15.3–60.6	33.5±13.1	32.7±11.4
4	25	12^th^ floor	5.1–54.0	27.4±9.4	24.5±7.0
5	26	12^th^ floor	10.6–43.0	26.3±12.7	26.5±11.1
6	26	12^th^ floor	10.1–85.4	66.8±21.7	23.7±6.1
7	24	12^th^ floor	4.5–54.3	21.5±13.2	16.9±6.7
8	30	6^th^ floor	4.9–49.1	24.5±13.1	25.8±12.4
9	25	6^th^ floor	5.5–204.0	28.0±13.6	30.9±12.6
10	26	6^th^ floor	10.7–99.2	21.9±14.5	24.6±10.8
11	28	1^st^ floor	10.0–137.0	39.8±13.7	22.8±8.2
12	31	1^st^ floor	10.2–114.5	40.6±16.1	22.3±10.9
13	29	1^st^ floor	4.5–84.4	35.0±9.2	18.7±7.8
14	28	1^st^ floor	4.8–58.0	23.6±9.3	19.7±8.4
15	26	1^st^ floor	4.0–221.0	18.5±6.7	16.8±8.7
16	24	1^st^ floor	4.7–86.4	46.4±10.0	14.3±7.8
17	27	1^st^ floor	4.4–59.0	28.2±6.2	24.2±10.3
18	28	1^st^ floor	4.0–93.3	21.3±7.9	19.2±7.4
19	26	1^st^ floor	4.2–88.5	30.1±12.1	18.4±8.3
20	28	1^st^ floor	5.0–55.0	22.5±6.9	19.4±7.7
21	30	1^st^ floor	4.0–119.3	38.0±11.6	21.3±7.4
Total average level			4.0–221.0	30.4±12.3	22.4±11.6

### 3.3 ^222^Rn and ^220^Rn concentrations on the different floors

The floor-specific 24- and 12-hour average ^222^Rn and ^220^Rn concentrations continuously measured in the 21 households are summarized in Table 3. The 24-hour average ^222^Rn concentrations in the bedrooms on the first, sixth, and twelfth floors were 42.2, 34.2, and 26.0 Bq/m^3^, respectively. The statistical analysis results demonstrated that significant differences existed between the floors (H = 118.504, *p*<0.0001). The 24-hour average ^222^Rn concentrations decreased in the following order: first floor > sixth floor > twelfth floor. The 12-hour average ^222^Rn concentrations in the bedrooms on the first, sixth, and twelfth floors were 44.2, 36.8, and 23.4 Bq/m^3^, respectively. The statistical analysis results showed that significant differences existed between the floors (H = 90.104, *p*<0.0001). The 12-hour average ^222^Rn concentrations also decreased in the following order: first floor > sixth floor > twelfth floor.

**Table 3:** Average ^222^Rn and ^220^Rn levels in the bedrooms on the different floors (Bq/m^3^).

Location	Number of rooms	Monitoring days	Average ^222^Rn level		Average ^220^Rn level	
			24-hour	12-hour	24-hour	12-hour
12^th^ floor	7	182	26.0±10.7	23.4±9.6	31.3±13.7	24.5±11.5
6^th^ floor	3	81	34.2±12.5	36.8±11.9	24.8±10.8	27.1±11.2
1^st^ floor	11	305	42.2±14.5	44.2±13.9	31.3±10.7	19.7±9.6

The 24-hour average ^220^Rn concentrations in the bedrooms on the first, sixth, and twelfth floors reached 31.1, 24.8, and 31.3 Bq/m^3^, respectively. The statistical analysis results indicated that there was no significant difference between the floors (H = 3.383, *p*>0.05). The 24-hour average ^220^Rn concentrations in the bedrooms on the first and twelfth floors were slightly higher than those in the bedrooms on the sixth floor. The 12-hour average ^220^Rn concentrations in the bedrooms on the first, sixth, and twelfth floors were 19.7, 27.1, and 24.5 Bq/m^3^, respectively. The statistical analysis results revealed that significant differences occurred between the floors (H = 19.780, *p*<0.001).

### 3.4 Variation trend of the ^222^Rn and ^220^Rn concentrations

**Fig 1:**
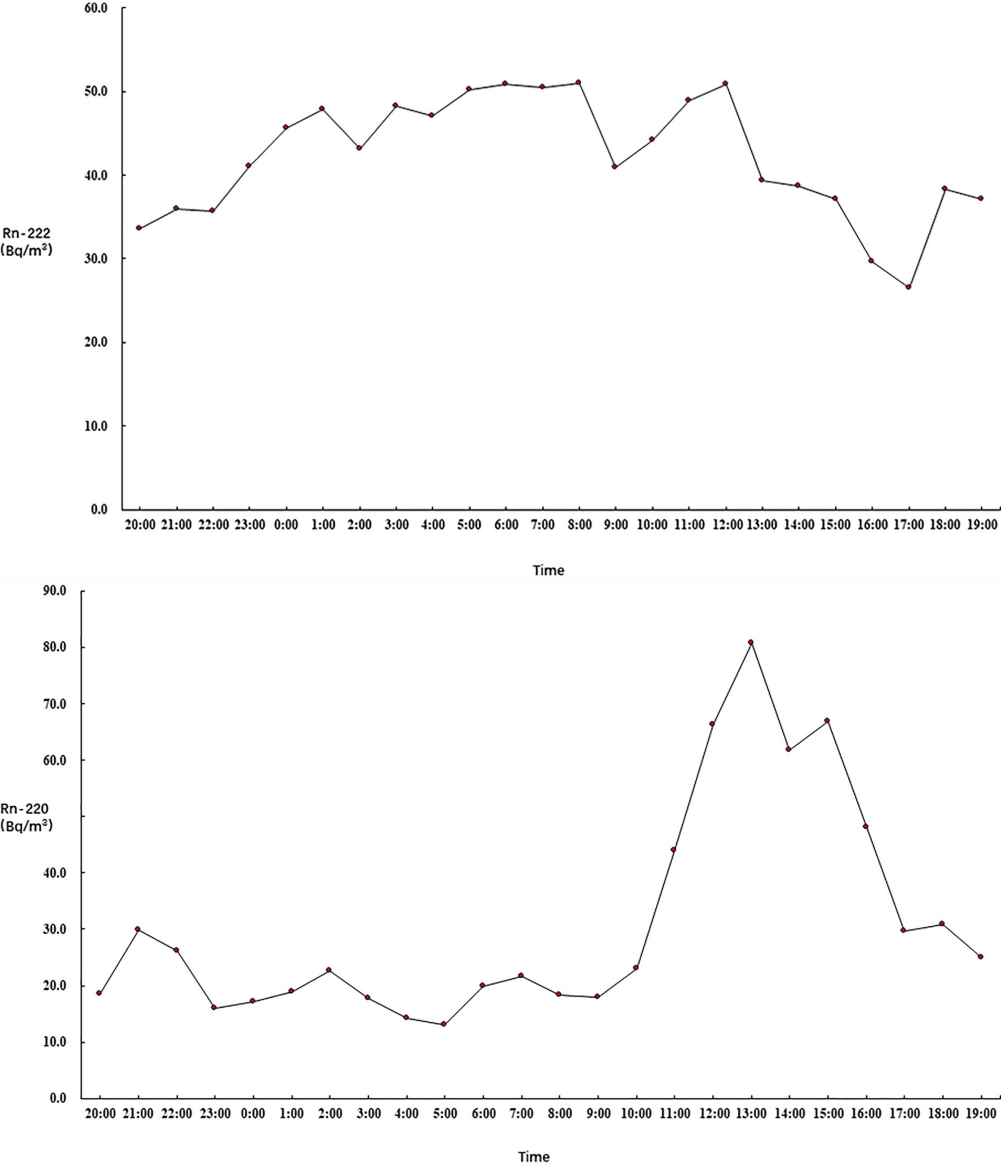
Radon variation on the first floor.

**Fig 2:**
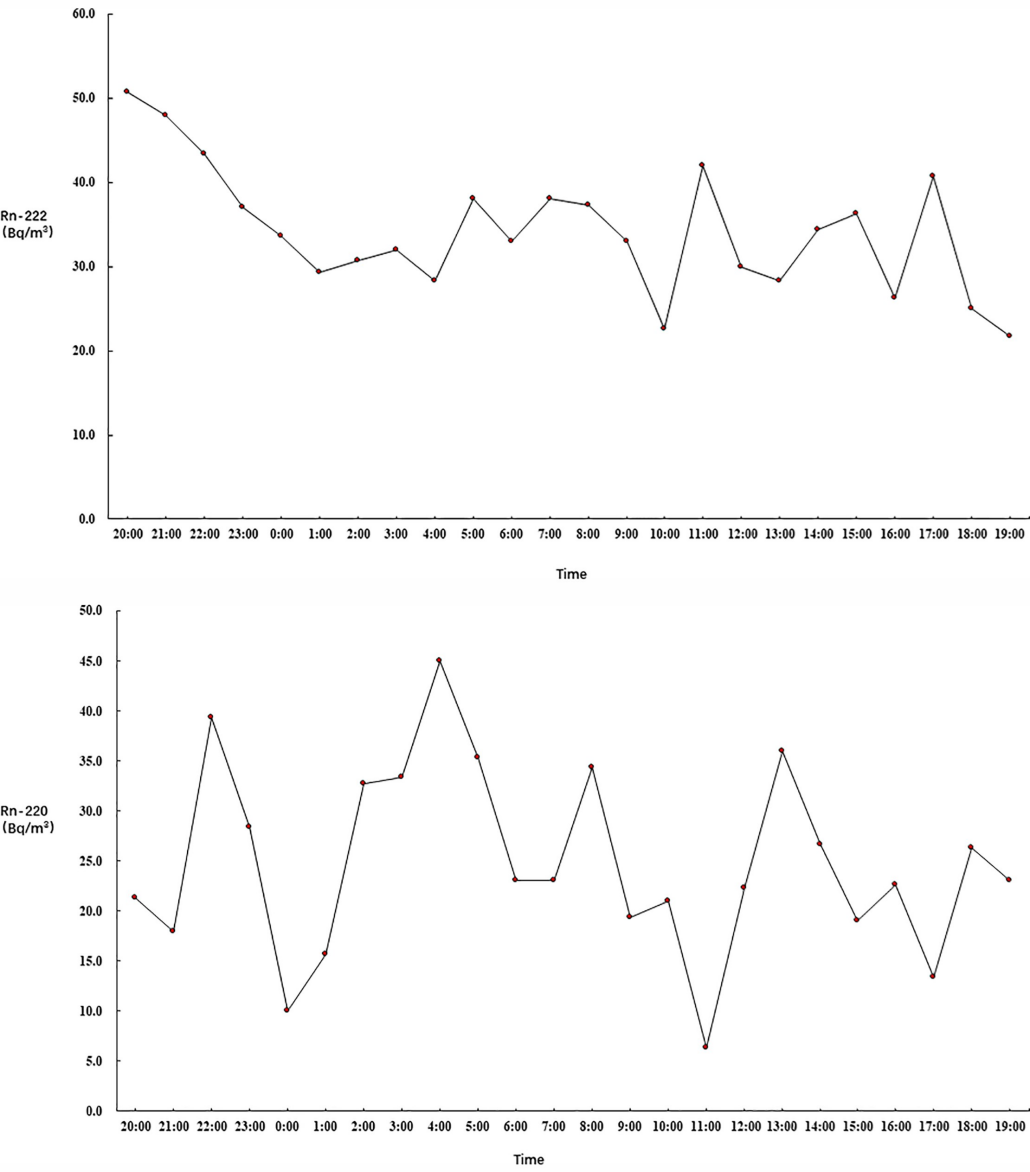
Radon variation on the sixth floor.

**Fig 3:**
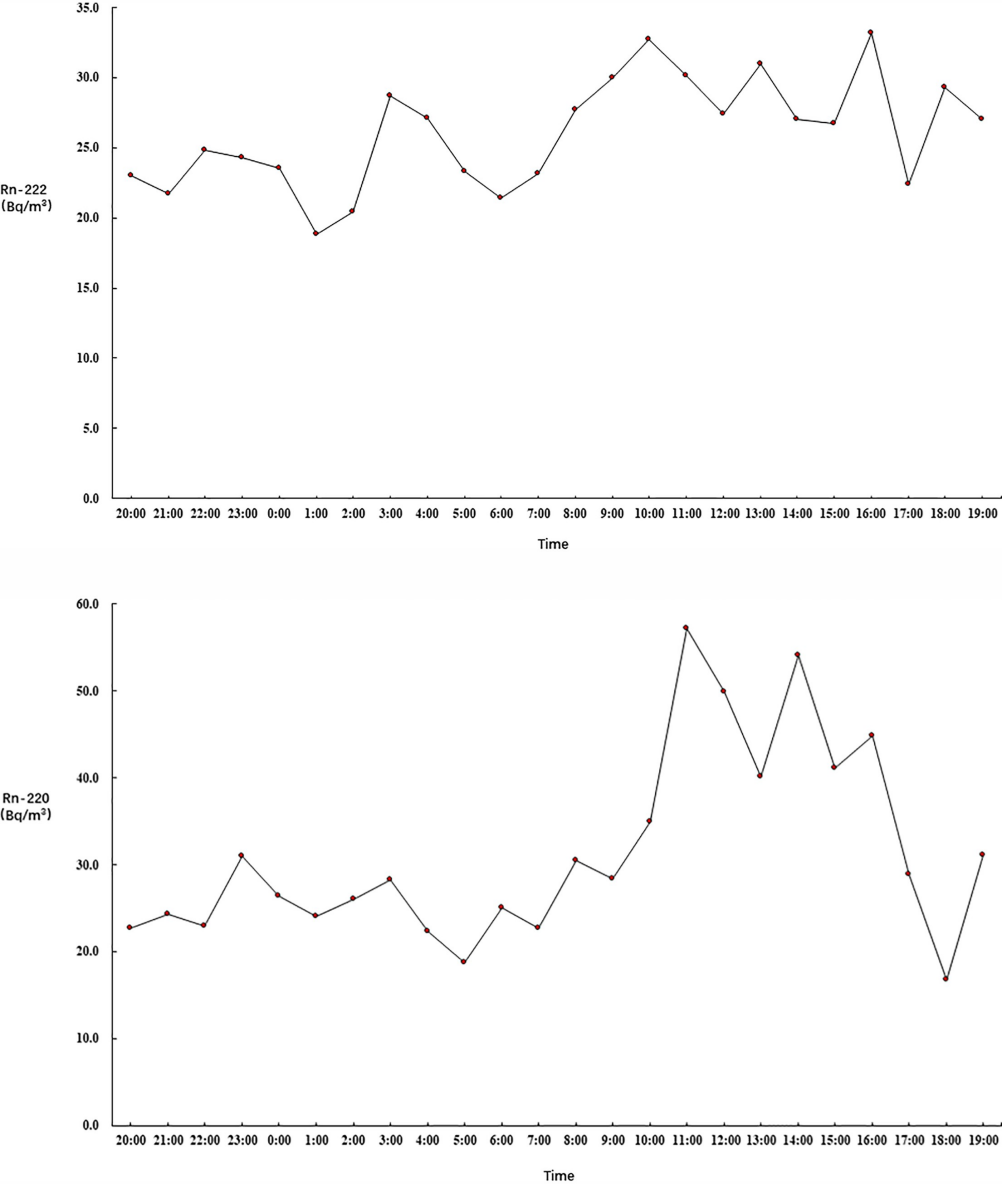
Radon variation on the twelfth floor.

Figs 1–3 show the 24-hour variation in the ^222^Rn and ^220^Rn concentrations in the 21 selected bedrooms on the different floors. Figs 1–3 show that the ^222^Rn and ^220^Rn concentrations fluctuated on the different floors during the detection period, but there was no obvious change trend.

### 3.5 Annual effective radiation dose estimation

In this study, our detection was conducted during the heating period. In northern China, the heating time usually starts on October 15 and continues to March 15 of the next year. During the heating season, ordinary residents often close doors and windows to reduce natural ventilation due to their needs for energy savings and heat preservation. This may lead to the accumulation of radon in residential environments. Hence, ^222^Rn and ^220^Rn may reach relatively high levels. To protect the health of residents, the average ^222^Rn and ^220^Rn concentrations in winter were considered as a representation of the annual average concentrations in residential environments to estimate the annual effective radiation dose for local residents. The dose estimation results are listed in Table 4.

**Table 4:** Annual effective radiation dose of radon and thoron in the different environments.

	C_R_ (Bq/m^3^)	D_R_ (mSv/y)	C_T_ (Bq/m^3^)	D_T_ (mSv/y)	Total annual effective dose (mSv/y)
Indoor residential environment	36.2	0.5978	22.4	0.4032	1.0010
Indoor working environment	29.5*	0.3215	29.5*	0.3505	0.6720
Outdoor environment	5.1*	0.0286	2.9**	0.0177	0.0463
Total annual effective dose (mSv/y)	-	0.9479	-	0.7714	1.7193

* reference [13], and

** reference [14].

## 4. Discussion

### 4.1 ^222^Rn in the residential environment and effective dose estimation

In this study, the 24- and 12-hour average ^222^Rn concentrations in the selected 21 bedrooms reached 35.7 and 36.2 Bq/m^3^, respectively. The maximum ^222^Rn concentration was approximately 85.0 Bq/m^3^. These results are far below the relevant criteria in the national indoor air quality standard in China (400 Bq/m^3^) and lower than the limit set by the WHO (100 Bq/m^3^). A nationwide environmental radon survey conducted from 1984 to 1990 in China showed that the national arithmetic mean ^222^Rn concentration was 22.5 Bq/m^3^, and in Shandong Province, the arithmetic mean concentration reached 18.7 Bq/m^3^ [15]. The results of investigations conducted in Japan [16] and South Korea [17] indicated that the arithmetic mean indoor ^222^Rn concentrations were 14.3 and 70.8 Bq/m^3^, respectively. Compared to the above domestic and foreign survey results, the indoor average ^222^Rn concentration in the residential environment in Weifang is higher than that in Shandong Province, China and Japan but lower than that in South Korea. The variation in these results is related to many factors, such as regional differences, sample sizes, detection methods, and weather conditions.

Statistical analysis revealed that there was no significant difference between the 24- and 12-hour average ^222^Rn concentrations in this study, which indicates that 12-hour monitoring data can be adopted to evaluate exposure levels rather than 24-hour monitoring data. In this study, the 12-hour average ^222^Rn concentration in winter was regarded as the annual average ^222^Rn level in Weifang city to calculate the annual effective radiation dose of ^222^Rn and its progeny for local ordinary residents. The average annual effective radiation dose of ^222^Rn exposure in the indoor residential environment was 0.5978 mSv. We also retrieved data from other studies to estimate the total average annual effective radiation dose for the local general population. Our results showed that the total average annual effective radiation dose of ^222^Rn for the general population in Weifang is approximately 0.9479 mSv, which is lower than that in two villages of Hengyang city (0.786 mSv) [18] based on a survey conducted in China. Previous studies have shown that the indoor radon concentrations in rooms on different floors exhibited significant differences [19, 20]. Floor-specific differences in ^222^Rn concentrations were observed in this study, suggesting that the floor should be considered in the estimation of individual ^222^Rn exposure levels.

### 4.2 ^220^Rn in the residential environment and effective dose estimation

In this study, the 24- and 12-hour average ^220^Rn concentrations in the 21 bedrooms were 30.4 and 22.4 Bq/m^3^, respectively. The maximum and minimum ^220^Rn values in the bedrooms reached approximately 221.0 and 4 Bq/m^3^, respectively. China has not yet set a limit for ^220^Rn in the national indoor air quality standard. The 24- and 12-hour average ^220^Rn concentrations in this study were lower than those detected in Beijing (56.4 Bq/m^3^), Guangzhou (190.8 Bq/m^3^), Zhuhai (127.9 Bq/m^3^), and Pingliang (493.5 Bq/m^3^) [21].

There was a significant difference between the 24- and 12-hour average ^220^Rn concentrations in this study according to the conducted statistical analysis, indicating that the detection duration may affect the average ^220^Rn level. The 12-hour average ^220^Rn concentration in winter was considered to calculate the annual effective radiation dose because the time truly spent in the residential environment is 12 hours. According to Eq (2), we obtained an average annual effective radiation dose of ^220^Rn of 0.4032 mSv in the indoor residential environment in Weifang city for ordinary residents. We also acquired data from other studies to calculate the total annual effective radiation dose of ^220^Rn, which was approximately 0.7714 mSv, which is much higher than that in two villages of Hengyang city (0.0185 mSv) in China [18]. Floor-specific differences in the ^220^Rn concentration were also observed, suggesting that the floor must be considered in the estimation of individual ^220^Rn exposure levels.

According to a report released by the UNSCEAR in 1998, the average annual effective radiation dose caused by the inhalation of radon and its progeny is approximately 1.2 mSv, accounting for approximately 50% of the total natural effective radiation dose of 2.4 mSv. Based on the data in this survey, the estimated annual effective radiation dose received by ordinary residents in Weifang city is 1.7193 mSv, of which 0.9479 mSv originates from ^222^Rn and its progeny and 0.7714 mSv originates from ^220^Rn and its progeny, accounting for 55.1% and 44.9%, respectively, of the total dose. Our findings suggest that ^220^Rn should not be ignored by local residents in Weifang city, and more attention should be paid to ^220^Rn in future research.

Inevitably, our findings have limitations. First, due to the condition and funding constraints, we chose 21 bedrooms in winter for on-the-spot detection. The small sample size and short detection time may lead to a limited representativeness of the test data. Second, this survey only used the adopted RAD7 radon detector to detect gaseous ^222^Rn and ^220^Rn but not their daughters. When estimating the annual effective radiation dose due to the progenies of ^222^Rn and ^220^Rn, conversion factors were considered, which involves a certain uncertainty.

## 5. Conclusions

Despite the limitations of our study, we obtained the following findings:

1. Through this field investigation, we preliminarily determined the ^222^Rn and ^220^Rn levels in the residential environment of Weifang city. The 24- and 12-hour average ^222^Rn levels were 35.7±15.2 Bq/m^3^ and 36.2±15.8 Bq/m^3^, respectively. The 24- and 12-hour average ^220^Rn levels reached 30.4±12.3 Bq/m^3^ and 22.4±11.6 Bq/m^3^, respectively.
2. Regardless of ^222^Rn or ^220^Rn, there were significant differences in the average level between floors.
3. The ^222^Rn and ^220^Rn levels in the residential environment fluctuated during the detection period, but no obvious long-term change trend was observed.
4. It was found in this study that the annual effective radiation dose of ^220^Rn for local residents is relatively high, suggesting that ^220^Rn should not be ignored, and more attention should be paid to ^220^Rn in future research.

## Supporting information

S1 Table Average ^222^Rn level in the 21 bedrooms (Bq/m^3^). (DOCX)

S2 Table Average ^220^Rn level in the 21 bedrooms (Bq/m^3^). (DOCX)
