# Supplementary material for: Investigation of the indoor 222Rn and 220Rn levels in the residential environment and estimation of the annual effective radiation dose for ordinary residents
Source: PLoS One. 2021 Jun 24;16(6):e0253463. doi: 10.1371/journal.pone.0253463 (PMC8224870; doi:10.1371/journal.pone.0253463)
Supplement: S1 Table — (DOCX) [file pone.0253463.s001.docx]

**S1 Table. Average ^222^R**n **level in the 21 bedrooms (Bq/m^3^)**

| Number of rooms | Monitoring days | Location | Range | 24-h average level | 12-h average level |
| --- | --- | --- | --- | --- | --- |
| 1 | 30 | 12^th^ floor | 9.2-39.5 | 20.4±6.9 | 20.0±8.5 |
| 2 | 25 | 12^th^ floor | 12.3-43.0 | 28.0±13.2 | 21.7±10.1 |
| 3 | 26 | 12^th^ floor | 10.5-43.2 | 24.8±11.1 | 20.7±9.7 |
| 4 | 25 | 12^th^ floor | 14.0-42.4 | 27.8±14.7 | 21.0±12.6 |
| 5 | 26 | 12^th^ floor | 3.0-41.7 | 25.5±11.1 | 19.9±9.2 |
| 6 | 26 | 12^th^ floor | 9.0-41.2 | 26.5±10.0 | 30.8±9.0 |
| 7 | 24 | 12^th^ floor | 10.6-55.9 | 29.2±12.4 | 29.5±11.3 |
| 8 | 30 | 6^th^ floor | 25.1-63.6 | 37.8±11.2 | 42.5±10.1 |
| 9 | 25 | 6^th^ floor | 20.0-53.1 | 30.8±13.2 | 27.9±14.0 |
| 10 | 26 | 6^th^ floor | 28.2-67.0 | 34.0±12.4 | 40.1±10.2 |
| 11 | 28 | 1^st^ floor | 40.9-72.3 | 49.7±13.0 | 52.3±11.8 |
| 12 | 31 | 1^st^ floor | 21.4-66.5 | 49.3±14.8 | 54.0±9.0 |
| 13 | 29 | 1^st^ floor | 28.2-63.6 | 43.1±13.2 | 41.5±13.8 |
| 14 | 28 | 1^st^ floor | 20.0-62.1 | 41.5±10.3 | 41.3±10.3 |
| 15 | 26 | 1^st^ floor | 21.6-56.4 | 37.2±15.7 | 41.6±14.4 |
| 16 | 24 | 1^st^ floor | 16.0-70.0 | 35.3±11.0 | 39.0±7.7 |
| 17 | 27 | 1^st^ floor | 15.2-66.0 | 45.3±16.2 | 45.8±16.9 |
| 18 | 28 | 1^st^ floor | 23.1-58.4 | 37.5±16.3 | 34.2±14.1 |
| 19 | 26 | 1^st^ floor | 22.5-70.0 | 40.2±13.4 | 44.3±13.2 |
| 20 | 28 | 1^st^ floor | 27.2-62.8 | 40.1±15.8 | 45.7±16.2 |
| 21 | 30 | 1^st^ floor | 26.1-85.0 | 45.1±13.6 | 46.3±15.4 |
| Total average level | | | 3.0-85.0 | 35.7±15.2 | 36.2±15.8 |
