# Supplementary material for: Investigation of the indoor 222Rn and 220Rn levels in the residential environment and estimation of the annual effective radiation dose for ordinary residents
Source: PLoS One. 2021 Jun 24;16(6):e0253463. doi: 10.1371/journal.pone.0253463 (PMC8224870; doi:10.1371/journal.pone.0253463)
Supplement: S2 Table — (DOCX) [file pone.0253463.s002.docx]

**S2 Table. Average ^220^Rn level in the 21 bedrooms (Bq/m^3^)**

| Number of rooms | Monitoring days | Location | Range | 24-h average level | 12-h average level |
| --- | --- | --- | --- | --- | --- |
| 1 | 30 | 12^th^ floor | 5.0-49.2 | 21.5±10.4 | 21.5±10.2 |
| 2 | 25 | 12^th^ floor | 10.1-44.5 | 22.4±9.0 | 26.0±8.7 |
| 3 | 26 | 12^th^ floor | 15.3-60.6 | 33.5±13.1 | 32.7±11.4 |
| 4 | 25 | 12^th^ floor | 5.1-54.0 | 27.4±9.4 | 24.5±7.0 |
| 5 | 26 | 12^th^ floor | 10.6-43.0 | 26.3±12.7 | 26.5±11.1 |
| 6 | 26 | 12^th^ floor | 10.1-85.4 | 66.8±21.7 | 23.7±6.1 |
| 7 | 24 | 12^th^ floor | 4.5-54.3 | 21.5±13.2 | 16.9±6.7 |
| 8 | 30 | 6^th^ floor | 4.9-49.1 | 24.5±13.1 | 25.8±12.4 |
| 9 | 25 | 6^th^ floor | 5.5-204.0 | 28.0±13.6 | 30.9±12.6 |
| 10 | 26 | 6^th^ floor | 10.7-99.2 | 21.9±14.5 | 24.6±10.8 |
| 11 | 28 | 1^st^ floor | 10.0-137.0 | 39.8±13.7 | 22.8±8.2 |
| 12 | 31 | 1^st^ floor | 10.2-114.5 | 40.6±16.1 | 22.3±10.9 |
| 13 | 29 | 1^st^ floor | 4.5-84.4 | 35.0±9.2 | 18.7±7.8 |
| 14 | 28 | 1^st^ floor | 4.8-58.0 | 23.6±9.3 | 19.7±8.4 |
| 15 | 26 | 1^st^ floor | 4.0-221.0 | 18.5±6.7 | 16.8±8.7 |
| 16 | 24 | 1^st^ floor | 4.7-86.4 | 46.4±10.0 | 14.3±7.8 |
| 17 | 27 | 1^st^ floor | 4.4-59.0 | 28.2±6.2 | 24.2±10.3 |
| 18 | 28 | 1^st^ floor | 4.0-93.3 | 21.3±7.9 | 19.2±7.4 |
| 19 | 26 | 1^st^ floor | 4.2-88.5 | 30.1±12.1 | 18.4±8.3 |
| 20 | 28 | 1^st^ floor | 5.0-55.0 | 22.5±6.9 | 19.4±7.7 |
| 21 | 30 | 1^st^ floor | 4.0-119.3 | 38.0±11.6 | 21.3±7.4 |
| Total average level | | | 4.0-221.0 | 30.4±12.3 | 22.4±11.6 |
